# Increased perivascular space volume in white matter and basal ganglia is associated with cognition in Parkinson’s Disease

**DOI:** 10.1007/s11682-023-00811-4

**Published:** 2023-10-19

**Authors:** Erin Kaye Donahue, Ryan Patrick Foreman, Jared Joshua Duran, Michael Walter Jakowec, Joseph O’Neill, Andrew J. Petkus, Daniel P. Holschneider, Jeiran Choupan, John Darrell Van Horn, Siva Venkadesh, Ece Bayram, Irene Litvan, Dawn M Schiehser, Giselle Maria Petzinger

**Affiliations:** 1 Department of Neurology, Keck School of Medicine, University of Southern California, 1333 San Pablo St, MCA-243, Los Angeles, CA 90033 USA; 2 Division of Child Psychiatry, UCLA Semel Institute for Neuroscience, Los Angeles, CA 90024 USA; 3 Department of Psychiatry & the Behavioral Sciences, University of Southern California, Los Angeles, CA 90033 USA; 4 Laboratory of NeuroImaging, USC Stevens Neuroimaging and Informatics Institute, Keck School of Medicine, University of Southern California, Los Angeles, CA 90033 USA; 5 Department of Psychology, University of Virginia, Charlottesville, VA 22904 USA; 6 School of Data Science, University of Virginia, Charlottesville, VA 22904 USA; 7 Parkinson and Other Movement Disorder Center, Department of Neurosciences, University of California San Diego, La Jolla, CA 92093 USA; 8 Veterans Administration San Diego Healthcare System (VASDHS), San Diego, CA 92161 USA; 9 Department of Psychiatry, University of California, San Diego, CA 92093 USA

**Keywords:** Virchow-Robinson space, Global cognition, Visuospatial function, Memory, Rostral middle frontal

## Abstract

Perivascular spaces (PVS), fluid-filled compartments surrounding brain vasculature, are an essential component of the glymphatic system responsible for transport of waste and nutrients. Glymphatic system impairment may underlie cognitive deficits in Parkinson’s disease (PD). Studies have focused on the role of basal ganglia PVS with cognition in PD, but the role of white matter PVS is unknown. This study examined the relationship of white matter and basal ganglia PVS with domain-specific and global cognition in individuals with PD. Fifty individuals with PD underwent 3T T1w magnetic resonance imaging (MRI) to determine PVS volume fraction, defined as PVS volume normalized to total regional volume, within (i) centrum semiovale, (ii) prefrontal white matter (medial orbitofrontal, rostral middle frontal, superior frontal), and (iii) basal ganglia. A neuropsychological battery included assessment of global cognitive function (Montreal Cognitive Assessment, and global cognitive composite score), and cognitive-specific domains (executive function, memory, visuospatial function, attention, and language). Higher white matter rostral middle frontal PVS was associated with lower scores in both global cognitive and visuospatial function. In the basal ganglia higher PVS was associated with lower scores for memory with a trend towards lower global cognitive composite score. While previous reports have shown that greater amount of PVS in the basal ganglia is associated with decline in global cognition in PD, our findings suggest that increased white matter PVS volume may also underlie changes in cognition.

**Supplementary Information:**

The online version contains supplementary material available at 10.1007/s11682-023-00811-4.

## Introduction

Parkinson’s disease (PD) is a progressive neurodegenerative disorder affecting both motor and nonmotor functions including cognition (Aarsland et al., 2017). Mild cognitive impairment (MCI) is common in PD, and can affect multiple cognitive domains, including executive function, attention, memory, and visuospatial function (Aarsland et al., 2021). MCI often progresses to dementia. Pathophysiological changes underlying cognitive changes remain poorly elucidated one potential mechanism may be due to dysfunction of the glymphatic system, leading to decreased clearance of metabolic waste products and protein aggregates from the brain parenchyma (Debette et al., 2019; Ding et al., 2017; Zhu et al., 2010).

In the healthy brain the glymphatic system serves several essential roles including (i) immune surveillance, (ii) transport of neurotransmitters and nutrients (e.g., glucose and lipids), and (iii) clearance of cellular debris, metabolic waste products, and protein aggregates (Jessen et al., 2015). A key component of the glymphatic system is the perivascular spaces (PVS), which are the fluid filled compartments surrounding perforating vasculature throughout the brain including the basal ganglia and white matter. PVS are bordered on one side by blood vessels and on the other side by astrocytic endfeet (Engelhardt & Ransohoff, 2012). Dysfunction of the glymphatic system has been reported as increased PVS volume or count, due to enlargement as determined by neuroimaging (Donahue et al., 2021).

Within the field of aging, studies have shown an association between greater amount of PVS and cognitive decline in both white matter and basal ganglia (MacLullich et al., 2004; Paradise et al., 2021). In PD, studies have also shown an association between increased basal ganglia PVS count and decreased cognitive performance. For example, higher PVS count is associated with worsening of global cognition as assessed by the Montreal Cognitive Assessment (MoCA). Additionally, in PD higher basal ganglia PVS count is associated with conversion from normal cognition to MCI, and from MCI to dementia (Chen et al., 2022; Park et al., 2019). While general cognition is associated with PVS in PD, the relationship between changes in PVS and deficits in specific cognitive domains has not been explored. Additionally, while there is an association between increased basal ganglia PVS and cognition, less is known about the relationship between changes in white matter PVS volume and cognition in PD.

The primary goal of this study was to evaluate PVS volume, defined as volume fraction, within overall and regional frontal white matter and the basal ganglia and its association with global and domain-specific cognitive performance. PVS volume was determined from T1w magnetic resonance imaging (MRI) using an automated method that quantitates PVS volume relative to total white matter volume in the local volume-of-interest (volume fraction) (Sepehrband et al., 2019). This allowed assessment of PVS within the large centrum semiovale white matter region of interest, as well as in selective regions of the frontal white matter (medial orbitofrontal, rostral middle frontal, superior frontal white matter) and the basal ganglia. The centrum semiovale and basal ganglia are regions in which PVS has been routinely abundant in past studies. Frontal lobe regions were selected for analysis due to its known role in cognitive function in PD. A full neuropsychological assessment was collected on study participants, including global cognition (MoCA and global composite score) and executive function, episodic memory (memory) visuospatial function, attention, and language scores, as well as assessment of cognitive status.

## Methods

### Study participants

For this cross-sectional analysis, data of 50 participants with idiopathic PD were obtained. Participants were evaluated as part of a multi-site (University of Southern California (USC) and Veterans Affairs San Diego Healthcare System (VASDHS) / University of California San Diego (UCSD) study. PD diagnosis was based on the UK Brain Bank criteria (Hughes et al., 1992). Inclusion criteria included: (i) willing and able to provide informed consent; (ii) medically eligible/safe for MRI; and (iii) Hoehn and Yahr score ≤ 3. Exclusion criteria included (i) history of severe psychiatric illness or other neurological disease, including prior stroke and (ii) dementia per criteria in Hoops et al., 2009 with MOCA < 18. Institutional Review Boards from each site approved all study procedures. Demographic data were collected, including age, sex, years of education, years of Parkinson’s disease diagnosis, MDS-Unified Parkinson’s Disease Rating Scale (MDS-UPDRS) part III score (motor), body mass index (BMI; kg/m^2^), Levodopa Equivalent Dosage (LED; mg) (*Parkinson’s Disease Measurement: PwP, Surveys, Trials, Analysis*, n.d.), and cardiovascular risk score (calculated by summing 0 = no/1 = yes for smoking, hypertension, high cholesterol, diabetes). Assessments were conducted in the ‘ON’ medication state.

### MRI acquisition

Structural MRI was acquired on a Siemens Prisma (USC) or a GE MR 750 3T MRI (UCSD) 3T MRI scanner. Whole-brain T1w MRI was acquired with a magnetization-prepared rapid gradient-echo pulse-sequence. On the Prisma the acquisition parameters were: flip 8°, repetition-time/echo-time/inversion-time = 2400/2.22/1000ms, voxels 0.8 × 0.8 × 0.8 mm3; 1 NEX, acceleration factor 2, 6:38 min; on the MR 750 the acquisition parameters were: flip 8°, repetition-time/echo-time/inversion-time = 2852/3.548/1000ms, voxels 0.8 × 0.8 × 0.8 mm3; 1 NEX, acceleration factor 2, 7:58 min. Study site was included as a covariate to account for any differences due to slight parameter changes.

### Perivascular space mapping

T1w MRI underwent preprocessing and regional parcellation with FreeSurfer v5.3.0. Preprocessing included motion correction, non-uniform intensity normalization, Talairach transformation, and skull stripping. PVS was mapped from T1w MRI as previously described (Donahue et al., 2021). T1w MRI was enhanced using an adaptive nonlocal mean filtering technique applied only on high-frequency spatial noise using a moving patch with a radius of one voxel (Manjón et al., 2010). A Frangi filter was applied using the Quantitative Imaging Toolkit (Cabeen, 2020; Frangi et al., 1998). A ‘vesselness’ probability measure was estimated for each voxel from eigenvectors of the Hessian matrix (parameter c set to half the value of the maximum Hessian norm) of the image at different scales (0.1–5 voxels) to provide a maximum likelihood/maximize vessel inclusion. This results in a brain-wide quantitative map of vesselness. A vesselness threshold of 0.0002 was used to obtain the PVS mask.

All T1w MRI were manually checked for misalignment, post-processing failure, lacunes, white-matter hyperintensities, which were corrected on manual inspection (Sepehrband et al., 2021; Benjamin et al., 2018). Brain volume and centrum semiovale, basal ganglia and frontal regional masks were derived using the Desikan-Killiany atlas (Desikan et al., 2006), using the *recon-all* module of Freesurfer. Centrum semiovale PVS was calculated by summing caudal middle frontal, inferior parietal, pars opercularis, pars orbitalis, pars triangularis, post central, precentral, rostral middle frontal, superior frontal, superior parietal, supramarginal, and unsegmented white matter not defined in the Desikan-Killiany atlas from both hemispheres. Because pathophysiological changes in the basal ganglia and frontal lobe are thought to be involved in early cognitive changes in PD, we also included these regions in our analysis (Farina et al., 2000; Lewis et al., 2003; Owen et al., 1995; Taylor et al., 1990). Frontal white matter regions included the medial orbitofrontal, the rostral middle frontal, and the superior frontal regions (note - rostral middle frontal and superior frontal are subregions of centrum semiovale). PVS volume measurements within each region were normalized to the total white matter volume (or total basal ganglia volume) of the same region to provide an internal control for possible PD-related changes in white matter volume. This produced a “volume fraction” that represents the fraction of the region of interest that is composed of PVS. All PVS results are expressed as volume fraction.

### Neuropsychological assessment

The neuropsychological battery measured global cognitive performance and performance across cognitive domains including executive function, memory, visuospatial function, attention, and language. Executive function was measured by (i) Wisconsin Card Sorting Test-64 card version (WCST) perseverative responses, (ii) Inhibition (condition 3) and Inhibition/Switching (condition 4) subtests from the Delis Kaplan Executive Function System (D-KEFS) Color Word Interference Test (CWIT), and (iii) D-KEFS Verbal Fluency Switching subtest. Memory was measured by (i) California Verbal Learning Test (2nd Edition; CVLT-II) Total Trials 1–5, Short-Delay Free Recall, and Long-Delay Free Recall, (ii) Brief Visuospatial Memory Test (BVMT) Immediate- and Delayed-Recall conditions. Visuospatial function was measured by the total scores on the (i) Judgment of Line Orientation Test and (ii) Hooper Visual Organization Test. Attention was measured by (i) D-KEFS CWIT Color Naming (Condition 1) and Word Reading (Condition 2) subtests and (ii) Adaptive Digit Ordering Test Forward Digit Span and Digit Sequencing conditions. Language was measured by (i) D-KEFS Verbal Fluency Letter Fluency and Category Fluency subtests and (ii) the Boston Naming Test. Domain scores were calculated by averaging the z-scores of all components. Global cognition was assessed using a mean of the five cognitive domain z-scores (Global Cognition). The MoCA score was used as a general cognition screen.

### Statistics

All statistical analyses were conducted using SPSS, Version 28.0.1 (Armonk, NY: IBM Corp). To be retained as significant (p ≤ 0.05), potential relationships between cognitive scores and regional PVS volume fraction had to survive both screening with a simple correlation analysis and a follow-up linear regression. A bivariate unadjusted Pearson correlation was performed between each cognitive domain score, and Global Cognition and MoCA scores, and the PVS volume fraction in each brain region (centrum semiovale, medial orbitofrontal, rostral middle frontal, superior frontal, and basal ganglia). If a correlation was significant, a hierarchical linear regression analysis using backwards elimination was applied (Lilja & Linse, 2022) to increase the power of comparisons, using: age, sex, years of education, years of Parkinson’s disease diagnosis, MDS-UPDRS part III score, BMI, LED, cardiovascular risk score, and study site (1 = USC/Siemens scanner, n = 21, and 2 = UCSD/GE scanner, n = 30). Regression p-values are Benjamini-Hochberg False Discovery Rate corrected within each predictor variable, to account for multiple comparisons (*False Discovery Rate Online Calculator | Tools*, n.d.). Any individual beyond three standard deviations in either the independent or dependent variable was considered an extreme outlier and was excluded from analyses for that comparison (3 removed from medial orbitofrontal, 1 removed from basal ganglia).

## Results

Participants were on average 65.36 years of age (SD = 8.98) with 16.68 (SD = 2.20) years of education; 54% were male, with a mean MDS-UPDRS part III score (motor) of 20.84 (SD = 9.08), mean Body Mass Index (BMI) of 25.89 kg/m^2^ (SD = 4.75), and a mean cardiovascular risk score of 0.56 (SD = 0.73) (Table 1). Mean scores for the individual cognitive tests used to calculate domain-specific z scores and MOCA scores are shown in Supplementary Table 1.

**Table 1:** Study demographics

	Mean (SD)
N (# of females)	50 (23)
Age	65.36 (8.98)
Education (years)	16.68 (2.20)
Body Mass Index (kg/m^2^)	25.89 (4.75)
MDS-UPDRS part III score	20.84 (9.08)
Years of Parkinson’s diagnosis	4.52 (5.34)
Cardiovascular Risk Score	0.56 (0.73)
Levodopa Equivalent Daily Dose (mg)	575.57 (394.01)

MDS-UPDRS: Movement Disorder Society-Unified Parkinson’s Disease Rating Scale

**Fig. 1:**
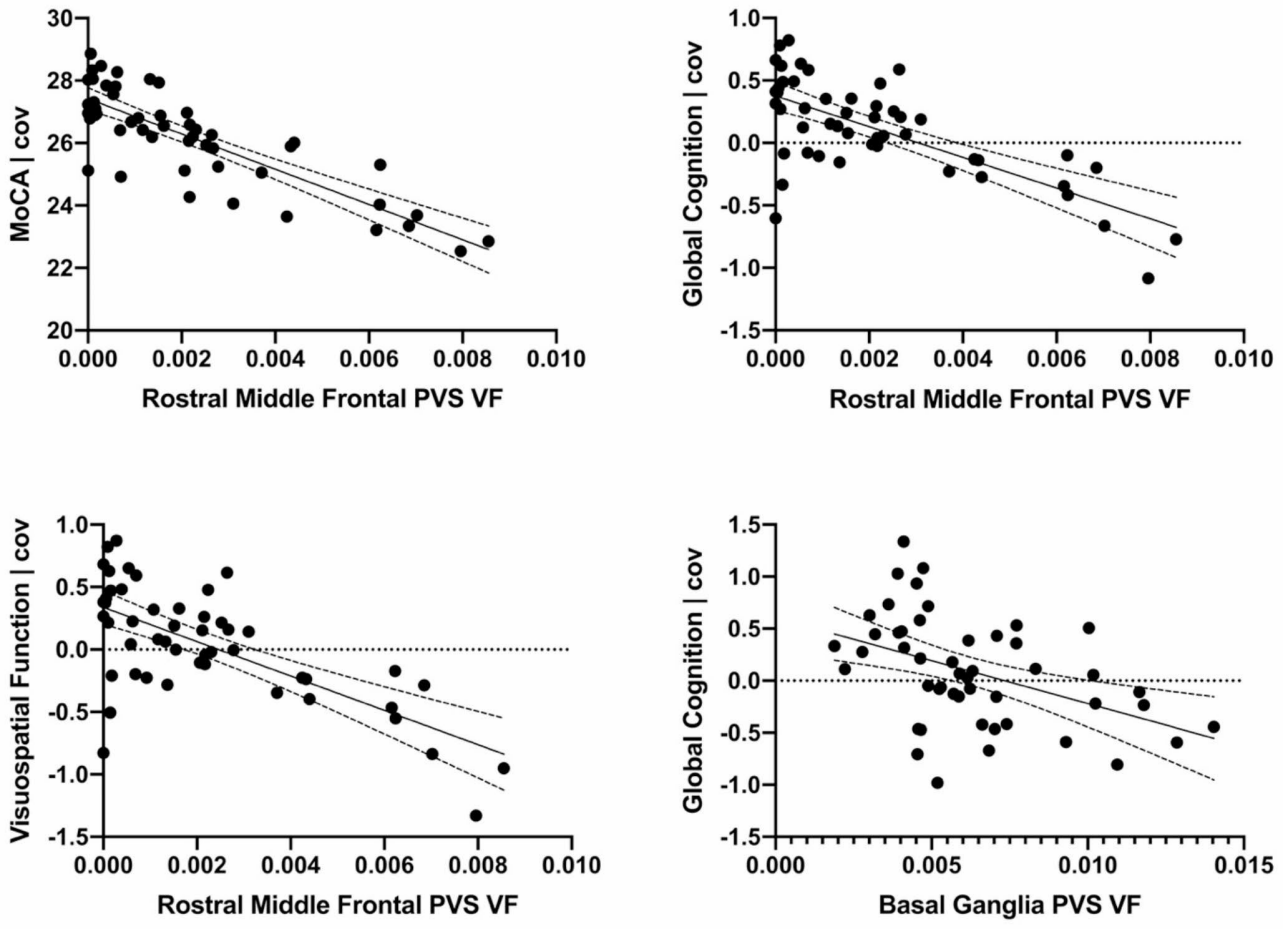
Significant adjusted associations between rostral middle frontal and basal ganglia perivascular space volume fraction and cognition. See Methods for covariates. PVS: perivascular space; VF: volume fraction

Rostral middle frontal PVS volume fraction was negatively correlated with (i) MoCA (*r*(48) = -0.524, *p* < 0.001), (ii) global cognition z score (*r*(48) = -0.380, p = 0.007), (iii) visuospatial function (*r*(48) = -0.336, *p* = 0.017), and (iv) memory (*r*(48) = -0.490, *p* < 0.001) (Supplementary Table 2). After adjusting for covariates in the linear regression, rostral middle frontal PVS volume fraction remained significantly associated with (i) MoCA (age, BMI; unstandardized β= -318.40, 95% CI [-613.85, -22.95], ΔR^2^ = 0.061, *p* = 0.047), (ii) global cognition (MDS-UPDRS part III score; unstandardized β= -112.22, 95% CI [-192.59, -31.84], ΔR^2^ = 0.119, *p* = 0.028), and (iii) visuospatial function (MDS-UPDRS part III score; unstandardized β= -124.79, 95% CI [-229.90, -19.67], ΔR^2^ = 0.092, *p* = 0.042), but not memory (age, sex, MDS-UPDRS part III score, site; unstandardized β= -51.43, 95% CI [-151.76, 48.91], ΔR^2^ = 0.009, *p* = 0.307) (Table 2; Fig. 1).

**Table 2:** Adjusted associations between perivascular space volume fraction in centrum semiovale, rostral middle frontal, and superior frontal white matter and the basal ganglia

	β	95% CI	*p*
		Lower, Upper	
**MoCA**			
centrum semiovale PVS	-73.84	-288.02, 140.34	0.982
rostral middle frontal PVS	-318.40*	**-613.85, -22.95**	**0.047**
superior frontal PVS	-80.33	-205.88, 45.22	0.408
basal ganglia PVS	-109.02	-321.32, 103.28	0.306
**Global Cognition**			
rostral middle frontal PVS	-112.22*	**-192.59, -31.84**	**0.028**
basal ganglia PVS	-84.65*	**-147.55, -21.76**	**0.030**
**Visuospatial Function**			
rostral middle frontal PVS	-124.79*	**-229.90, -19.67**	**0.042**
**Memory**			
centrum semiovale PVS	-16.22	-88.46, 56.01	0.653
rostral middle frontal PVS	-51.43	-151.76, 48.91	0.307
superior frontal PVS	1.26	-42.91, 45.42	0.954
basal ganglia PVS	-57.99	-127.88, 11.90	0.153

PVS: perivascular space

Significant regressions in **bold**. *p < 0.05, **p < 0.01, ***p < 0.001

Basal ganglia PVS volume fraction was negatively correlated with (i) MoCA (*r*(47) = -0.313, *p* = 0.029), (ii) global cognition z-score (*r*(47) = -0.311, p = 0.029), and (iii) memory (*r*(47) = -0.308, *p* = 0.031) (Supplementary Table 2). After adjusting for covariates in the linear regression, basal ganglia PVS volume fraction remained significantly associated with global cognition z-score (age, sex, MDS-UPDRS part III score, years of education; unstandardized β= -84.65, 95% CI [-147.55, -21.76], ΔR^2^ = 0.087, *p* = 0.030) but not memory (age, sex, MDS-UPDRS part III score, site; unstandardized β= -57.99, 95% CI [-127.88, 11.90], ΔR^2^ = 0.021, *p* = 0.153) or MoCA (age, sex, BMI, site; unstandardized β= -109.02, 95% CI [-321.32, 103.28], ΔR^2^ = 0.012, *p* = 0.306) (Table 2; Fig. 1).

Centrum semiovale PVS volume fraction was negatively associated with MoCA (*r*(48) = -0.358, *p* = 0.011) and memory (*r*(48) = -0.374, *p* = 0.007). Associations did not remain significant after adjusting for covariates: centrum semiovale PVS volume fraction-MoCA (age, BMI, site; unstandardized β= -73.84, 95% CI [-288.02, 140.34], ΔR^2^ = 0.006, *p* = 0.982), and centrum semiovale PVS volume fraction-memory (age, sex, MDS-UPDRS part III score, site; unstandardized β= -16.22, 95% CI [-88.46, 56.01], ΔR^2^ = 0.002, *p* = 0.653) (Table 2).

Superior frontal PVS volume fraction was negatively associated with MoCA (*r*(48) = -0.337 p = 0.017) and memory (*r*(48) = -0.293, p = 0.039). Associations did not remain significant after adjusting for covariates; superior frontal PVS volume fraction-MoCA (age, BMI, site; unstandardized β= -85.33, 95% CI [-205.88, 45.22], ΔR^2^ = 0.021, *p* = 0.408), and superior frontal PVS volume fraction-memory (age, sex, MDS-UPDRS part III score, site; unstandardized β = 1.26, 95% CI [-42.91, 45.42], ΔR^2^ = 0.000, *p* = 0.954) (Table 2).

No associations were observed between medial orbitofrontal PVS volume fraction and cognitive function. No associations were observed in any region between PVS volume fraction and language, attention, or executive function.

## Discussion

The aim of this study was to investigate the association of increased PVS volume in white matter and basal ganglia with domain-specific and global cognitive performance in PD. This study identified a significant association between increased rostral middle frontal PVS volume and general cognition as measured by MoCA and a global cognitive composite score in individuals with PD. Given that our average MOCA score was 26.18 it is possible that our findings were driven by individuals with mild cognitive impairment and will be investigated in future studies with a larger cohort. In addition to rostral middle frontal volume, there was a significant association between basal ganglia PVS volume and a global cognitive composite score. The rostral middle frontal region is located within the prefrontal cortex, encompassing both the dorsolateral and anterior prefrontal cortex. While a few studies have demonstrated an association between basal ganglia PVS and global cognition in PD, our study is the first to demonstrate a relationship between white matter PVS volume and both global and domain specific cognitive performance in PD (Chen et al., 2022; Park et al., 2019). Our methodological approach assessed change in large diameter PVS (considered > 3 mm) and excluded lacunes (small PVS) (Ramirez et al., 2022). Future studies will examine if both small and large diamgter PVS are associated with cognition in PD (Ramirez et al., 2022). This association of global cognition with rostral middle frontal and basal ganglia PVS may be due to the known contribution of both brain regions in early PD-related cognitive changes (Lewis et al., 2003). We also observed a relationship between increased rostral middle frontal white matter PVS volume and worse visuospatial function. A previous study showed an association between centrum semoivale white matter PVS and changes in visuospatial function in healthy aging (MacLullich et al., 2004). Our study aligns with this finding in that PVS in the rostral middle frontal white matter, a segment of the centrum semiovale, was associated with our visuospatial function composite. Further, studies have demonstrated a relationship between the rostral middle frontal region and tests of visuospatial function. Specifically, fMRI studies have shown activation in rostral middle frontal areas, including the dorsolateral and anterior prefrontal cortex, in individuals conducting the Hooper visual organization task (HVOT; Moritz et al., 2004), which is a visuospatial test with an executive function component.

While a few studies have previously reported an association between basal ganglia PVS and cognition in PD, to the best of our knowledge, our study is the first to report that white matter PVS may also contribute to cognitive performance in PD (Chen et al., 2022; Park et al., 2019). One possible explanation for this discrepancy may be due to differences in methodology for examining PVS burden. In our study, PVS was assessed as volume (volume fraction), as opposed to PVS counts or a ranking system, as reported in other studies (Chen et al., 2022; Park et al., 2019). Volumetric quantification may capture a more complete picture of PVS involvement in disease pathophysiology given that a significant characteristic of PVS dysfunction is thought to be dilation (Zou et al., 2019). For example, PVS volume in white matter has been shown to be higher in PD relative to healthy controls (Donahue et al., 2021). Taken together, our results and previous studies suggest that PVS volume changes could contribute to PD pathophysiology, including cognitive performance.

Currently, the mechanisms leading to increased PVS amount, and cognitive decline are unknown. One suggestion is that PVS volume may reflect decreased flow in the glymphatic system resulting in either increased accumulation of metabolic waste products or failure to deliver needed metabolic substrates. For example, surgical block of glymphatic flow in rodents leads to the accumulation of alpha-synuclein, a pathophysiological protein hallmark of PD (Zou et al., 2019). Glymphatic flow may also be disrupted by chronic inflammatory processes know to occur in many neurodegenerative disorders including PD (de Groot & Burgas, 2015; De Virgilio et al., 2016; Klein, 2000; Mogensen et al., 2021). Changes in PVS may also be associated with reduced integrity of the blood brain barrier (BBB) (Chen et al., 2021). For example, a recent study from our group showed an association between increased PVS volume in the frontal white matter and increased choline-containing compounds a potential marker of astrocyte gliosis (Donahue et al., 2022). Since astrocytes are critical component of BBB structure and function, these changes may reflect BBB breakdown or reduced neuroenergetic support by astrocytes, both impacting glymphatic integrity reflected by PVS pathology (Urenjak et al., 1993). Further studies are needed to identify the underlying mechanisms leading to increased PVS in disorders of the brain including PD and how such changes lead to disease state and progression.

Understanding the impact of changes in PVS in PD and its progression are important since studies have suggested a strong association between lifestyle factors (sleep, exercise) and glymphatic system function. Animal studies show that glymphatic flow increases during sleep (Xie et al., 2013). Clinical studies have demonstrated an association between increased PVS and obstructive sleep apnea and longer self-reported time in bed (Jia et al., 2021; Ramirez et al., 2021). Furthermore, voluntary running has also been shown to increase glymphatic flow and clearance of amyloid-beta protein aggregates in rodents (He et al., 2017; von Holstein-Rathlou et al., 2018). These reports highlight the potential targeting of the glymphatic systems through lifestyle that may impact disease progression in PD including cognitive decline.

### Limitations and strengths

There are several limitations to this study. First, the absence of T2-weighted images precluded using the automated mapping pipeline to fully discern PVS from white matter hyperintensities and lacunes. Instead, all PVS masks were quality control checked to remove any white matter hyperintensities or lacune erroneously mapped as PVS. Second, the cross-sectional design of the study prevents the determination of causality in the relationship between PVS and cognition. Third, this study did not examine the independent contribution of cerebrovascular burden to cognition. However, given that cardiovascular risk profile is related to cerebrovascular burden, and our participants have very low cardiovascular risk profiles (mean of ~ 0.5 score on 5 yes/no questions) and no history of stroke, supports that PVS may be an important contributor to cognitive performance independent of cerebrovascular burden. Future longitudinal studies will examine longitudinal relationships between PVS and cognition to determine directionality. There are several strengths of this study including (i) utilization of automated (objective) assessment of PVS, and (ii) determination of PVS volume rather than count may provide insight into mechanisms of glymphatic dysfunction.

## Conclusions

This study supports that greater PVS volume in either white matter or basal ganglia is associated with worse cognitive performance in PD. Understanding PVS pathophysiology may provide insight into function of the glymphatic system and its impact on PD and its progression.

### Electronic supplementary material

Below is the link to the electronic supplementary material.

Supplementary Material 1

## Data Availability

Data available upon request to GMP.

## References

[CR2] Aarsland D, Creese B, Politis M, Chaudhuri KR, Ffytche DH, Weintraub D, Ballard C (2017). Cognitive decline in Parkinson Disease. Nature Reviews Neurology.

[CR1] Aarsland D, Batzu L, Halliday GM, Geurtsen GJ, Ballard C, Chaudhuri R, Weintraub D (2021). Parkinson disease-associated cognitive impairment. Nature Reviews Disease Primers.

[CR3] Benjamin P, Trippier S, Lawrence AJ, Lambert C, Zeestraten E, Williams OA, Patel B, Morris RG, Barrick TR, MacKinnon AD, Markus HS (2018). Lacunar infarcts, but not perivascular spaces, are predictors of Cognitive decline in Cerebral Small-Vessel Disease. Stroke.

[CR4] Cabeen, R. P. (2020, May 6). *Quantitative Imaging Toolkit: Software for Interactive 3D Visualization, Data Exploration, and Computational Analysis of Neuroimaging Datasets*. Ryan Cabeen. /about/publication/cabeen-2018-quantitative/.

[CR6] Chen S, Shao L, Ma L (2021). Cerebral edema formation after Stroke: Emphasis on blood-brain barrier and the lymphatic drainage system of the brain. Frontiers in Cellular Neuroscience.

[CR5] Chen H, Wan H, Zhang M, Wardlaw JM, Feng T, Wang Y (2022). Perivascular space in Parkinson’s Disease: Association with CSF amyloid/tau and cognitive decline. Parkinsonism & Related Disorders.

[CR7] de Groot NS, Burgas MT (2015). Is membrane homeostasis the missing link between inflammation and neurodegenerative Diseases?. Cellular and Molecular Life Sciences: CMLS.

[CR8] De Virgilio A, Greco A, Fabbrini G, Inghilleri M, Rizzo MI, Gallo A, Conte M, Rosato C, Ciniglio Appiani M, de Vincentiis M (2016). Parkinson’s Disease: Autoimmunity and neuroinflammation. Autoimmunity Reviews.

[CR9] Debette S, Schilling S, Duperron MG, Larsson SC, Markus HS (2019). Clinical significance of magnetic resonance imaging markers of Vascular Brain Injury: A systematic review and Meta-analysis. JAMA Neurology.

[CR10] Desikan RS, Ségonne F, Fischl B, Quinn BT, Dickerson BC, Blacker D, Buckner RL, Dale AM, Maguire RP, Hyman BT, Albert MS, Killiany RJ (2006). An automated labeling system for subdividing the human cerebral cortex on MRI scans into gyral based regions of interest. Neuroimage.

[CR11] Ding J, Sigurðsson S, Jónsson PV, Eiriksdottir G, Charidimou A, Lopez OL, van Buchem MA, Guðnason V, Launer LJ (2017). Large perivascular spaces visible on magnetic resonance imaging, Cerebral Small Vessel Disease Progression, and risk of Dementia: The Age, Gene/Environment susceptibility–Reykjavik Study. JAMA Neurology.

[CR13] Donahue EK, Murdos A, Jakowec MW, Sheikh-Bahaei N, Toga AW, Petzinger GM, Sepehrband F (2021). Global and Regional changes in Perivascular Space in Idiopathic and Familial Parkinson’s Disease. Movement Disorders: Official Journal of the Movement Disorder Society.

[CR12] Donahue EK, Bui V, Foreman RP, Duran JJ, Venkadesh S, Choupan J, Van Horn JD, Alger JR, Jakowec MW, Petzinger GM, O’Neill J (2022). Magnetic resonance spectroscopy shows associations between neurometabolite levels and perivascular space volume in Parkinson’s Disease: A pilot and feasibility study. Neuroreport.

[CR14] Engelhardt B, Ransohoff RM (2012). Capture, crawl, cross: The T cell code to breach the blood-brain barriers. Trends in Immunology.

[CR15] *False Discovery Rate Online Calculator | Tools*. (n.d.). Retrieved December 19, (2022). from https://tools.carbocation.com/FDR.

[CR16] Farina E, Gattellaro G, Pomati S, Magni E, Perretti A, Cannatà AP, Nichelli P, Mariani C (2000). Researching a differential impairment of frontal functions and explicit memory in early Parkinson’s Disease. European Journal of Neurology.

[CR17] Frangi, A. F., Niessen, W. J., Vincken, K. L., & Viergever, M. A. (1998). *Multiscale vessel enhancement filtering*. 130–137.

[CR18] He X, Liu D, Zhang Q, Liang F, Dai G, Zeng J, Pei Z, Xu G, Lan Y (2017). Voluntary Exercise promotes glymphatic clearance of amyloid Beta and reduces the activation of astrocytes and microglia in aged mice. Frontiers in Molecular Neuroscience.

[CR19] Hoops S, Nazem S, Siderowf AD, Duda JE, Xie SX, Stern MB, Weintraub D (2009). Validity of the MoCA and MMSE in the detection of MCI and Dementia in Parkinson Disease. Neurology.

[CR20] Hughes AJ, Daniel SE, Kilford L, Lees AJ (1992). Accuracy of clinical diagnosis of idiopathic Parkinson’s Disease: A clinico-pathological study of 100 cases. Journal of Neurology Neurosurgery and Psychiatry.

[CR21] Jessen NA, Munk ASF, Lundgaard I, Nedergaard M (2015). The Glymphatic System: A beginner’s guide. Neurochemical Research.

[CR22] Jia Y, Liu C, Li H, Li X, Wu J, Zhao Y, Xu M, Yu H, Guan Z, Sun S, Zhang C, Duan Z (2021). Enlarged Perivascular Space and its correlation with Polysomnography indicators of obstructive sleep apnea. Nature and Science of Sleep.

[CR23] Klein J (2000). Membrane breakdown in acute and chronic neurodegeneration: Focus on choline-containing phospholipids. Journal of Neural Transmission (Vienna Austria: 1996).

[CR24] Lewis SJG, Dove A, Robbins TW, Barker RA, Owen AM (2003). Cognitive impairments in early Parkinson’s Disease are accompanied by reductions in activity in frontostriatal neural circuitry. The Journal of Neuroscience: The Official Journal of the Society for Neuroscience.

[CR25] Lilja, D. J., & Linse, G. M. (2022). *Linear Regression Using R: An Introduction to Data Modeling, 2nd Edition*. University of Minnesota Libraries Publishing. 10.24926/8668/1301.

[CR26] MacLullich AMJ, Wardlaw JM, Ferguson KJ, Starr JM, Seckl JR, Deary IJ (2004). Enlarged perivascular spaces are associated with cognitive function in healthy elderly men. Journal of Neurology Neurosurgery & Psychiatry.

[CR27] Manjón JV, Coupé P, Martí-Bonmatí L, Collins DL, Robles M (2010). Adaptive non-local means denoising of MR images with spatially varying noise levels. Journal of Magnetic Resonance Imaging: JMRI.

[CR28] Mogensen FLH, Delle C, Nedergaard M (2021). The Glymphatic System (En)during inflammation. International Journal of Molecular Sciences.

[CR29] Moritz CH, Johnson SC, McMillan KM, Haughton VM, Meyerand ME (2004). Functional MRI neuroanatomic correlates of the Hooper Visual Organization Test. Journal of the International Neuropsychological Society: JINS.

[CR30] Owen AM, Sahakian BJ, Semple J, Polkey CE, Robbins TW (1995). Visuo-spatial short-term recognition memory and learning after temporal lobe excisions, frontal lobe excisions or amygdalo-hippocampectomy in man. Neuropsychologia.

[CR31] Paradise M, Crawford JD, Lam BCP, Wen W, Kochan NA, Makkar S, Dawes L, Trollor J, Draper B, Brodaty H, Sachdev PS (2021). Association of Dilated Perivascular Spaces with Cognitive decline and Incident Dementia. Neurology.

[CR32] Park YW, Shin NY, Chung SJ, Kim J, Lim SM, Lee PH, Lee SK, Ahn KJ (2019). Magnetic resonance imaging-visible Perivascular spaces in basal Ganglia Predict Cognitive decline in Parkinson’s Disease. Movement Disorders: Official Journal of the Movement Disorder Society.

[CR33] *Parkinson’s Disease Measurement: PwP, surveys, trials, analysis*. (n.d.). Retrieved June 12, (2022). from https://www.parkinsonsmeasurement.org/toolBox/levodopaEquivalentDose.htm.

[CR35] Ramirez J, Holmes MF, Berezuk C, Kwan D, Tan B, Beaton D, Scott CJM, Ozzoude M, Gao F, Yu D, Swardfager W, Lawrence-Dewar J, Dowlatshahi D, Saposnik G, Boulos MI, Murray BJ, Symons S, Bartha R, Black SE, Lim A (2021). MRI-visible perivascular space volumes, sleep duration and daytime dysfunction in adults with Cerebrovascular Disease. Sleep Medicine.

[CR34] Ramirez J, Berberian SA, Breen DP, Gao F, Ozzoude M, Adamo S, ONDRI Investigators (2022). Small and large magnetic resonance imaging–visible Perivascular spaces in the basal ganglia of Parkinson’s Disease patients. Movement Disorders.

[CR36] Sepehrband F, Barisano G, Sheikh-Bahaei N, Cabeen RP, Choupan J, Law M, Toga AW (2019). Image processing approaches to enhance perivascular space visibility and quantification using MRI. Scientific Reports.

[CR37] Sepehrband F, Barisano G, Sheikh-Bahaei N, Choupan J, Cabeen RP, Lynch KM, Crawford MS, Lan H, Mack WJ, Chui HC, Ringman JM, Toga AW (2021). Volumetric distribution of perivascular space in relation to mild cognitive impairment. Neurobiology of Aging.

[CR38] Taylor AE, Saint-Cyr JA, Lang AE (1990). Memory and learning in early Parkinson’s Disease: Evidence for a frontal lobe syndrome. Brain and Cognition.

[CR39] Urenjak J, Williams SR, Gadian DG, Noble M (1993). Proton nuclear magnetic resonance spectroscopy unambiguously identifies different neural cell types. The Journal of Neuroscience: The Official Journal of the Society for Neuroscience.

[CR40] von Holstein-Rathlou S, Petersen NC, Nedergaard M (2018). Voluntary running enhances glymphatic influx in awake behaving, young mice. Neuroscience Letters.

[CR41] Xie, L., Kang, H., Xu, Q., Chen, M. J., Liao, Y., Thiyagarajan, M., O’Donnell, J., Christensen, D. J., Nicholson, C., Iliff, J. J., Takano, T., Deane, R., & Nedergaard, M. (2013). Sleep drives Metabolite Clearance from the adult brain. *Science (New York N Y)*, *342*(6156), 10.1126/science.1241224. 10.1126/science.1241224 PMC3880190 24136970

[CR42] Zhu YC, Dufouil C, Soumaré A, Mazoyer B, Chabriat H, Tzourio C (2010). High degree of dilated Virchow-Robin Spaces on MRI is Associated with increased risk of Dementia. Journal of Alzheimer’s Disease: JAD.

[CR43] Zou W, Pu T, Feng W, Lu M, Zheng Y, Du R, Xiao M, Hu G (2019). Blocking meningeal lymphatic drainage aggravates Parkinson’s disease-like pathology in mice overexpressing mutated α-synuclein. Translational Neurodegeneration.

