# Supplementary material for: Increased perivascular space volume in white matter and basal ganglia is associated with cognition in Parkinson’s Disease
Source: Brain Imaging Behav. 2023 Oct 19;18(1):57–65. doi: 10.1007/s11682-023-00811-4 (PMC10844402; doi:10.1007/s11682-023-00811-4)
Supplement: Supplementary file 1 — Supplementary Material 1 [file 11682_2023_811_MOESM1_ESM.docx]

**Supplementary Table 1:** *Neuropsychological testing scores in Parkinson’s disease patients*

| Language | Mean Score (SD) |
| --- | --- |
| DKEFS Letter Fluency | 43.16 (11.08) |
| DKEFS Category Fluency | 40.28 (8.95) |
| Boston Naming Test | 57.52 (2.85) |
| *Language Domain z-score* | 0.19361 (0.92596) |
| Attention |  |
| DKEFS CWIT Color Naming | 32.10 (5.37) |
| DKEFS CWIT Word Reading | 23.04 (4.59) |
| DOT-A Forward Digit Span | 7.94 (2.08) |
| DOT-A Digit Sequencing | 6.12 (2.36) |
| *Attention Domain z-score* | 0.08867 (0.93758) |
| Visuospatial Function |  |
| Judgment of Line Orientation | 23.82 (3.98) |
| Hooper Visual Organization Test | 24.92 (3.03) |
| *Visuospatial Function Domain z-score* | 0.03591 (0.95181) |
| Episodic Memory |  |
| CVLT-II Total Immediate Recall | 42.64 (10.05) |
| CVLT-II Short Delay Free Recall | 8.44 (3.51) |
| CVLT-II Long Delay Free Recall | 8.60 (3.64) |
| BVMT Immediate Recall | 16.36 (7.02) |
| BVMT Delayed Recall | 7.32 (3.35) |
| *Memory Domain z-score* | 0.09938 (1.02356) |
| Executive Function |  |
| DKEFS CWIT Color-Word Inhibition | 61.42 (13.79) |
| DKEFS CWIT Inhibition/Switching | 70.56 (31.25) |
| DKEFS Verbal Fluency/Switching | 13.40 (3.02) |
| WCST Perseverative Response | 9.46 (8.22) |
| *Executive Function Domain z-score* | 0.11084 (0.99584) |
| General Cognition |  |
| Montreal Cognitive Assessment | 26.18 (2.51) |
| *Global Cognition z-score* | 0.10568 (0.75202) |

BVMT: Brief Visuospatial Memory Test; CVLT-II: California Verbal Learning Test (2nd Edition); CWIT: Color Word Interference Test; DKEFS: Delis Kaplan Executive Function; DOT-A: Adaptive Digit Ordering Test; WCST: Wisconsin Card Sorting Test

**Supplementary Table 2:** *Pearson correlations between neuropsychological testing scores and regional volume fractions of perivascular space (PVS) in Parkinson’s disease patients*

|  | MoCA | | Global  Cognition | | Language | | Attention | | Visuospatial  Function | | Memory | | Executive  Function | |
| --- | --- | --- | --- | --- | --- | --- | --- | --- | --- | --- | --- | --- | --- | --- |
| PVS Volume  Fraction | **r** | ***p*** | **r** | ***p*** | **r** | ***p*** | **r** | ***p*** | **r** | ***p*** | **r** | ***p*** | **r** | ***p*** |
| centrum semiovale | **-0.358*** | **0.011** | -0.217 | 0.129 | -0.065 | 0.651 | -0.053 | 0.713 | -0.238 | 0.096 | **-0.374**** | **0.007** | -0.098 | 0.499 |
| basal ganglia | **-0.313*** | **0.029** | **-0.311*** | **0.029** | -0.250 | 0.084 | -0.169 | 0.246 | -0.229 | 0.114 | **-0.308*** | **0.031** | -0.244 | 0.091 |
| medial orbitofrontal | -0.216 | 0.144 | -0.145 | 0.331 | 0.043 | 0.775 | 0.032 | 0.831 | -0.247 | 0.095 | -0.249 | 0.092 | -0.134 | 0.370 |
| rostral middle frontal | **-0.524***** | **<0.001** | **-0.380**** | **0.007** | -0.194 | 0.176 | -0.199 | 0.167 | **-0.336*** | **0.017** | **-0.490***** | **<0.001** | -0.242 | 0.091 |
| superior frontal | **-0.337*** | **0.017** | -0.178 | 0.217 | -0.109 | 0.453 | -0.020 | 0.893 | -0.189 | 0.189 | **-0.293*** | **0.039** | -0.069 | 0.632 |

PVS: perivascular space

Significant correlations in **bold**. *p<0.05, **p<0.01, ***p<0.001

**Supplementary Figure 1:** *Regions of the Desikan Killiany atlas included in regional perivascular space assessment. The white matter underlying each of these regions was used in analysis.*

**
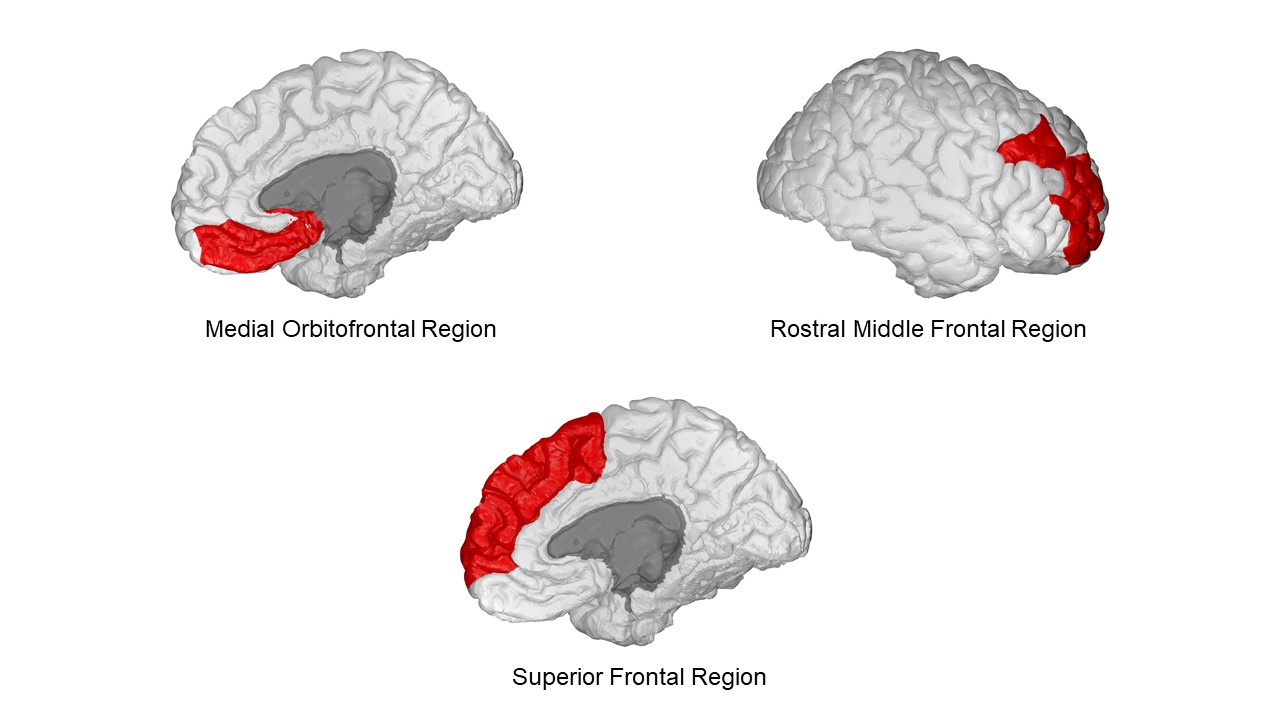
**
